# Sarcopenia and Malnutrition Screening in Female Osteoporosis Patients—A Cross-Sectional Study

**DOI:** 10.3390/jcm10112344

**Published:** 2021-05-27

**Authors:** Franca Genest, Dominik Rak, Elisa Bätz, Kerstin Ott, Lothar Seefried

**Affiliations:** 1 Clinical Trial Unit, Orthopedic Department, University of Würzburg, Brettreichstrasse 11, 97074 Wuerzburg, Germany; f-genest.klh@uni-wuerzburg.de (F.G.); d-rak.klh@uni-wuerzburg.de (D.R.); ott_k2@ukw.de (K.O.); 2 Institut für Medizinmanagement und Gesundheitswissenschaften, Universität Bayreuth, 95444 Bayreuth, Germany; elisa.baetz@web.de

**Keywords:** osteoporosis, malnourishment, sarcopenia, nutritional status, physical performance

## Abstract

Sarcopenia and malnutrition are important determinants of increased fracture risk in osteoporosis. SARC-F and MNA-SF are well-established questionnaires for identifying patients at risk for these conditions. We sought to evaluate the feasibility and potential added benefit of such assessments as well as the actual prevalence of these conditions in osteoporosis patients. We conducted a cross-sectional, single-center study in female osteoporosis patients ≥ 65 years (SaNSiBaR-study). Results of the sarcopenia (SARC-F) and malnutrition (MNA-SF) screening questionnaires were matched with a functional assessment for sarcopenia and data from patients’ medical records. Out of 107 patients included in the analysis, a risk for sarcopenia (SARC-F ≥ 4 points) and a risk for malnutrition (MNA-SF ≤ 11 points) was found in 33 (30.8%) and 38 (35.5%) patients, respectively. Diagnostic overlap with coincident indicative findings in both questionnaires was observed in 17 patients (16%). As compared to the respective not-at-risk groups, the mean short physical performance battery (SPPB) score was significantly reduced in both patients at risk for sarcopenia (7.0 vs. 10.9 points, *p* < 0.001) and patients at risk for malnutrition (8.7 vs. 10.5 points, *p* = 0.005). Still, confirmed sarcopenia according to EWGSOP2 criteria was present in only 6 (6%) of all 107 patients, with only 3 of them having an indicative SARC-F score. Bone mineral density was not significantly different in any of the at-risk groups at any site. In summary, applying SARC-F and MNA-SF in osteoporosis patients appears to be a complementary approach to identify individuals with functional deficits.

## 1. Introduction

Osteoporosis is an age-associated disease characterized by an increased fracture risk due to bone mineral density loss and microarchitectural deterioration of bone structure [1,2]. Fractures in the elderly are associated with adverse health outcomes including consecutive decline in physical activity and loss of mobility as well as increased morbidity and mortality [3,4]. While bone-targeted drugs used to improve bone mineral density and reduce fracture risk are the well-established mainstay of osteoporosis treatment, the therapeutic potential of identifying and modifying additional risk factors for fractures appears to be insufficiently recognized [5,6,7,8]. Two major risk factors readily amenable to therapeutic intervention are sarcopenia and malnutrition. Sarcopenia is the age-associated loss of muscle mass, strength and muscle function, leading to decreased mobility and increased fracture risk. In order to verbally convey this close correlation of sarcopenia and osteoporosis the term “osteosarcopenia” has been coined [9,10,11]. Growing evidence supports the perception that physical exercise can effectively counteract loss of muscle mass and function and associated fracture risk even in the oldest and weakest individuals [12,13,14]. Correspondingly, malnutrition and a lack of distinct macro- and micronutrients, particularly proteins, is known to have substantial impact on bone and muscle health, predisposing elderly patients to an increased risk for falls, fractures and mortality [15,16,17]. However, the only indicator of nutritional status currently applied in osteoporosis care is BMI. While there are data suggesting that both low BMI and underweight as well as elevated BMI and obesity affect BMD and fracture risk, data and evidence regarding the clinical significance and underlying mechanisms are very heterogeneous [18].

Since comprehensive diagnostic assessments for sarcopenia and malnutrition are complex and time consuming, respective evaluations have not been widely adopted in clinical routine. Recognizing this diagnostic gap, the European Working Group on Sarcopenia in Older People (EWGSOP) has recently updated their diagnostic algorithm to facilitate screening for sarcopenia by recommending the five item SARC-F questionnaire for case findings, and readily available testing procedures, i.e., the handgrip strength (HGS) or the chair rise test (CRT) to assess what is now termed “probable sarcopenia” in clinical practice. Quantitation of muscle mass and physical performance by measuring certain abilities, e.g., usual gait speed (UGS) or assessing the short physical performance battery (SPPB) are intended to confirm and grade severity of sarcopenia in a scientific setting [19].

Similarly, the MNA-SF has been widely used as a practicable and validated screening tool to identify malnourished older adults and those with a propensity for malnutrition in various settings [20], including its application in orthogeriatric patients [21].

In order to evaluate the feasibility and a potential added value of including both these screening questionnaires, SARC-F and MNA-SF, into the diagnostic workup of osteoporosis patients, this study aimed at evaluating the prevalence of sarcopenia and malnutrition as assessed by these questionnaires in osteoporosis patients, as well as to assess their information with regards to osteoporosis management and by matching the respective findings with additional diagnostic results.

## 2. Materials and Methods

### 2.1. Patients and Study Design

This is a single-center cross-sectional study in female patients ≥65 years of age with a confirmed diagnosis of osteoporosis and an indication for bone-targeted treatment according to applicable national osteoporosis guidelines [22]. Patients were excluded if they had to comply with dietary restrictions for other accompanying illnesses.

Following the provision of written informed consent, data regarding dual X-ray absorptiometry (DXA) scans and laboratory data were obtained from clinical routine medical records. Specifically, 25-Hydroxyvitmain D levels were assessed using the Elecsys^®^ Vitamin D total II assay (Roche Diagnostics InternationalAG, Rotkreuz, Switzerland).

### 2.2. Sarcopenia and Body Constitution

Assessment for sarcopenia was accomplished according to the European Working Group on Sarcopenia (EWGSOP2) guidelines [19] following the Find-Assess-Confirm-Severity (F-A-C-S) algorithm, starting with the SARC-F questionnaire, covering the 5 items of strength, assistance in walking, getting up from a chair, climbing stairs and falls for the case findings. The SARC-F assigns 0–2 points to each of the above items depending on severity. Patients attaining a total score of ≥4 out of 10 points should be further evaluated for sarcopenia [23]. Subsequent technical assessment for probable sarcopenia is based on the measurement of HGS or the CRT [10,24]. Grip strength measurement was accomplished using handheld isometric dynamometry (DynEx1, Akern srl, Florence, Italy) and a cut off for HGS of less than 16 kg was determined as deficient muscle strength [19]. CRT was measured by taking the time it takes for a patient to get up from a chair five times without using her hands and without using the arm rest. In case this takes > 15 s this is considered an indicator of probable sarcopenia.

Quantitation of muscle mass for confirmation of sarcopenia was accomplished in conjunction with comprehensive physical assessment comprising body height and weight as well as bioelectrical impedance analysis (BIA) (BIA 101 Anniversary, Akern SRL, Florence, Italy). The skeletal muscle index (SMI), i.e., skeletal muscle mass divided by height^2^ to adjust for body size, was calculated by applying the pre-established formula for the respective device [25,26,27]. Following the EWGSOP2 algorithm, low muscle mass is defined at an SMI ≤ 6.0 kg/m^2^, which is eventually deemed as confirmation of sarcopenia and was used in this study.

Additional deficits in physical performance, determined as 8 or less out of 12 points in the well-established SPPB, was based on integrative scoring of the balance test (BT), UGS over a 4 m stretch and the CRT. Alternatively, an isolated finding of reduced UGS < 0.8 m/s was defined as a hallmark of severe sarcopenia according to EWGSOP2.

### 2.3. Nutritional Assessment

Nutritional status was assessed using the Mini Nutritional Assessment-Short Form (MNA-SF) [20,28], a well-established standard questionnaire for identifying malnutrition in elderly people, including the items appetite, weight loss, mobility, acute diseases and stress, neuropsychological problems and either BMI or calf circumference. In this study, calf circumference was used. While a result of ≥12 out of max 14 points is considered adequate nutritional status, 8–11 points suggests a risk of malnutrition and subjects attaining ≤ 7 points are deemed malnourished. For purposes of this study, patients were subdivided into two groups with either adequate nutritional status (12–14 points) or a status at risk for or actual malnutrition (≤11 points) [20].

### 2.4. Statistical Analysis

The power calculation was based on an estimated prevalence of <10% for both sarcopenia and deficient nutrition (MNA), respectively. Accordingly, assessing about 100 participants was considered appropriate to allow for evaluating actual prevalence with a precision of ±6% or better (length of the 95% confidence interval).

Descriptive statistical analysis comprised absolute frequencies and corresponding proportions, arithmetic means and standard deviation. Correlation analyses were performed using Pearson’s correlation coefficient while between-group differences were assessed using independent samples’ *t*-tests. *p* values of less than 0.05 were considered statistically significant. All statistical analyses were performed using SPSS ver. 25 statistical software package (Released 2017, IBM SPSS Statistics for Windows, Version 25.0., IBM Corp., Armonk, NY, USA).

The study protocol (SaNSiBaR-study) was approved by the competent ethics committee at Würzburg University (270/17-me) and registered with the German register for clinical studies (DRKS00014296). Informed consent was obtained from all individual participants included in the study.

## 3. Results

**Figure 1:**
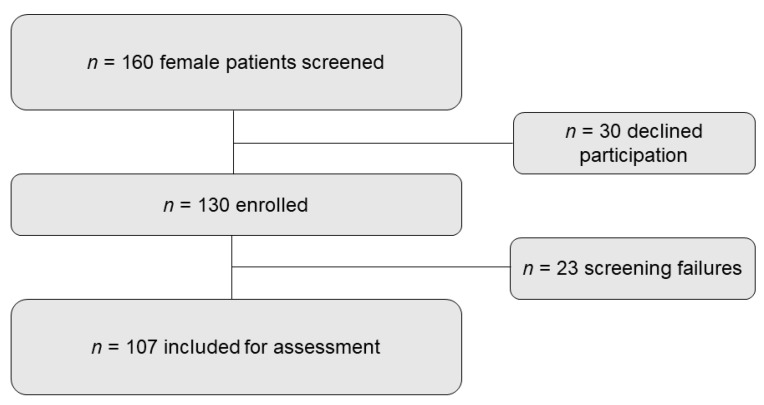
Flow diagram of study participants.

Out of 160 female patients screened, *n* = 130 were enrolled, of whom 27 did not meet the selection criteria, leaving 107 study participants to be assessed in this study (Figure 1).

Average age of these 107 women was 75.0 years (65–89 years, SD 5.9 years), mean values (SD) for height and weight were 158.8 cm (SD 6.0 cm) and 64.6 kg (SD 11.9 kg), respectively. Mean BMI was 25.6 kg/m^2^ (SD 4.4 kg/m^2^). Vitamin D levels were compensated and ≥20 ng/mL for all but one patient who was measured at 19 ng/mL. For details regarding patient characteristics see Table 1.

### 3.1. Prevalence of Sarcopenia

Screening for sarcopenia using the SARC-F questionnaire revealed ≥ 4 points and suspected sarcopenia in 33 patients (30.8%). Out of these, 9 patients had a HGS of < 16 kg and 6 more required > 15 s to perform the CRT, consistent with a diagnosis of probable sarcopenia in 15 patients of whom 3 exhibited an ASM/height^2^ < 6.0 kg/m^2^ eventually confirming sarcopenia. Two of them exhibited reduced UGS and one of the two had additionally reduced SPPB ≤ 8 points as indicators of severe sarcopenia.

Conversely, in the overall cohort, reduced HGS < 16 kg was documented in 15 patients and 12 more had a pathologic CRT result, i.e., following the EWGSOP2 algorithm, 27 were assessed to have probable sarcopenia, with 6 of them having reduced SMI < 6 kg/m^2^. Actual prevalence of confirmed sarcopenia was 6% (6/107) but only 3 of them were detected by SARC-F screening. Of interest, diminished SMI < 6.0 kg/m^2^ was observed in 13 subjects (12.1%) even though 7 of them, i.e., more than half of those with reduced muscle mass, did not have any pathologic findings concerning the SARC-F questionnaire, HGS, CRT, UGS or SPPB.

Still, comparative analyses of patients with inconspicuous and suspicious SARC-F results revealed that the latter were significantly older and exhibited significantly inferior physical performance results regarding HGS (*p* < 0.001), CRT (*p* = 0.004), UGS (*p* < 0.001) and SPPB (*p* < 0.001). In addition, patients with an indicative SARC-F score had significantly higher, i.e., worse MNA scores (*p* = 0.023). Interestingly, patients with suspicious SARC-F scores did not exhibit reduced muscle mass (SMI). Details are provided in Table 1.

### 3.2. Nutritional Status

In total, 8 out of 107 patients (7.5%) were underweight with a BMI < 20 kg/m^2^, while 14 (13%) had a BMI ≥ 30 indicating obesity. Analyzing the MNA-SF questionnaire *n* = 38 (35.5%) patients had a score ≤ 11 points and were either at risk to be malnourished (*n* = 33) or were actually malnourished (*n* = 5). Patients with a compromised nutritional status were significantly older (*p* = 0.035) and had a lower BMI (*p* = 0.005), even though only 5 out of these 38 had a BMI < 20 kg/m^2^. Patients with alleged malnutrition (MNA-SF ≤ 11) demonstrated significantly inferior physical performance, specifically regarding UGS (*p* = 0.003) and SPPB scores (*p* = 0.005). Indeed, 11 patients (10.3%) identified by MNA-SF screening demonstrated severely reduced physical performance with a SPPB score ≤ 8 points. Patients with supposed malnutrition according to MNA-SF also attained significantly higher, i.e., worse SARC-F scores (*p* = 0.006), suggesting a higher risk for sarcopenia. Results concerning CRT and HGS and even muscle mass (SMI) were not statistically significant between nutritional groups. Interestingly, 6 out of the 38 patients identified by inferior MNA-SF results had a low SMI, with 4 of them having confirmed sarcopenia.

### 3.3. Sarcopenic and Malnourished Patients

**Figure 2:**
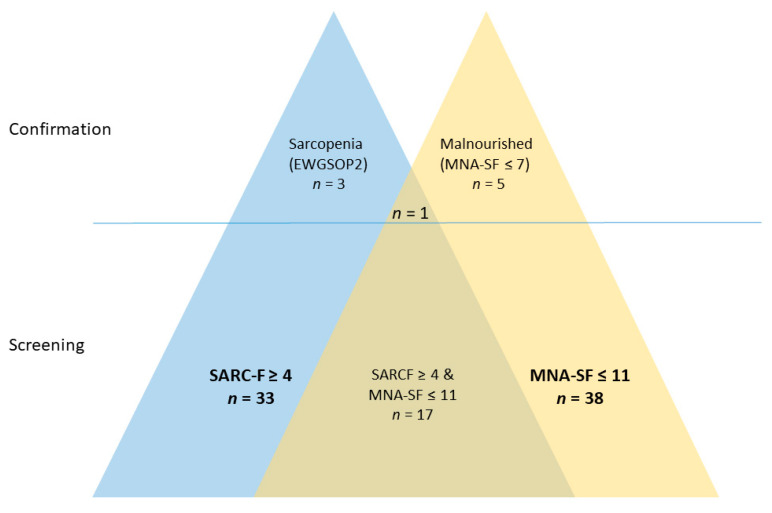
Number of patients with indicative findings in either or both of the two screening questionnaires (SARC-F and MNA-SF) and those with confirmed sarcopenia/malnutrition.

Out of these *n* = 107 female patients with osteoporosis, 54 (50.5%) demonstrated an indicative result in at least one of the two questionnaires but only 17 had coincident obvious findings in both, the SARC-F and the MNA-SF, i.e., the majority of 37 individuals had suggestive results in only one of the two screening tools, either the SARC-F (*n* = 16) or the MNA-SF (*n* = 21). In addition, there was only one patient with confirmed sarcopenia (SMI ≤ 6 kg/m^2^, HGS < 16 kg) and actual undernutrition (MNA-SF ≤ 7) (Figure 2).

### 3.4. Bone Mineral Density

According to the common concept of reduced bone mineral density (BMD) in patients with sarcopenia and malnutrition, we performed a comparative analysis of BMD in patients with and without obvious findings in the questionnaires under scrutiny.

**Figure 3:**
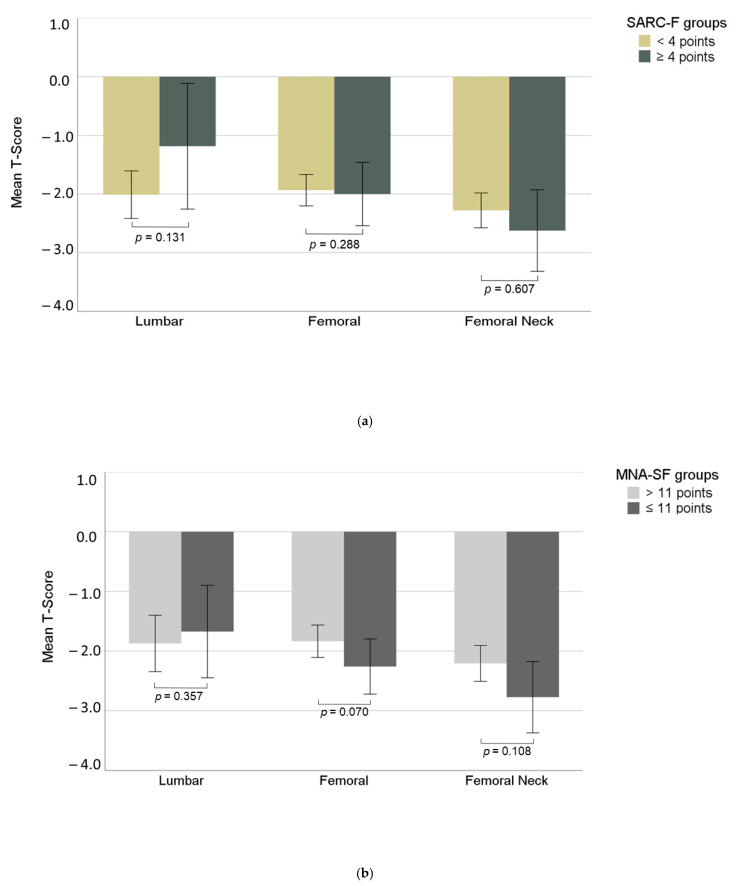
(**a,b**): differences between T-scores for different scores in MNA-SF and SARC-F questionnaires, respectively.

Dividing patient groups on the basis of normal or suspicious findings regarding the SARC-F or the MNA-SF score, respectively, we did not observe any significant differences regarding BMD values, neither at the lumbar spine nor at the femur (Figure 3).

## 4. Discussion

This study sought to evaluate a potential added value of including two well-established screening questionnaires for sarcopenia (SARC-F) and malnutrition (MNA-SF) into the routine diagnostic workup of osteoporosis patients. To that regard, results were matched with additional functional and constitutional outcome parameters to learn if the questionnaires help in identifying specific patient groups and how these can be characterized. Along with that, we aimed at quantitating the prevalence of both these conditions in a clinical routine cohort of elderly female patients with osteoporosis. Technically, answering the questionnaires was easily and quickly accomplished (about 10 min on average) by all patients without any difficulties. The overall prevalence of patients with suspected sarcopenia and malnutrition according to SARC-F and MNA-SF was 30.8% and 35.5%, respectively. In total, more than half of the patients (50.5%, *n* = 54) had an indicative result in at least one of the two questionnaires.

While this study used BIA to assess muscle mass, which could be considered a limitation, future approaches could include direct measurement of muscle mass using DXA along with DX measurements of muscle mass. Owing to national radiation protection regulations, the assessment of muscle mass was assessed by BIA instead of direct DXA measurement, which could be considered a limitation of our study.

Conversely, and in line with regional data on the prevalence of sarcopenia [29], only six patients (6%) in this cohort had confirmed sarcopenia according to EWGSOP2 of whom only three were detected with the SARC-F, which is actually in line with the well-known limited sensitivity (63%) and specificity (47%) of the SARC-F in terms of diagnosing sarcopenia [30].

Similarly, applying the lower MNA-SF cut at <8 points, only five patients (5%) were deemed malnourished. Notwithstanding the high sensitivity (88%) and specificity (87%) of this lower cut off point of the MNA-SF for predicting undernutrition [31], the larger group at ≤11 points would warrant extensive nutritional assessment, and immediate information for osteoporosis care remains elusive.

These findings have two major implications. First, the absolute prevalence of confirmed sarcopenia and the actual malnutrition in an outpatient cohort of osteoporosis patients according to criteria used here is quite low. Second, with regards to screening for patients to confirm sarcopenia and actual malnutrition, the absolute yield of applying these screening tools under scrutiny in clinical routine care for osteoporosis patients appears to be limited.

In contrast, previous studies confirm an association of inferior results in SARC-F [32,33] and MNA-SF [34,35,36] in those patients who do eventually experience fractures and who have a high risk for a poor rehabilitation outcome.

In line with that, our data unanimously confirm that indicative findings in either SARC-F or MNA-SF are associated with significantly compromised physical performance. While only few of those patients with indicative SARC-F results can be confirmed to be sarcopenic, they demonstrate significantly inferior muscle strength and physical performance regarding HGS, UGS, CRT and SPPB. This is actually in line with a previous study coming to the same conclusion that the validity of SARC-F in terms of screening for sarcopenia is limited, but it substantially improves the predictive value of predicting poor physical performance [37]. Similarly, even though we did not find remarkable constitutional peculiarities in those with indicative MNA-SF results, they also exhibited inferior physical performance, specifically concerning UGS and the SPPB. Importantly, there is only a limited overlap regarding the individuals identified with these two screening tools (Figure 2), i.e., they work in a largely complementary way with regards to identifying patients with compromised physical performance. In this combination, the questionnaires identified 50% of patients with compromised physical performance. Considering the proven clinical significance of poor physical performance, specifically for chair stand time walking speed and grip strength as known predictors of increased fracture risk [38,39], this appears critical in order to optimize fracture-preventive strategies. Indeed, the analysis of DXA data of those patients identified by SARC-F and MNA-SF screening confirmed that patients identified by means of these questionnaires do not have reduced BMD, i.e., their additional risk is not mirrored in low BMD but has to be recognized separately.

Sarcopenia and malnutrition are not reflected in low BMD and have to be considered as distinct aspects of fracture risk. Improving osteoporosis treatment by addressing these additional risk factors requires approaches distinct from bone-targeted drugs focusing on BMD and bone stability.

These findings support the perception that MNA-SF and SARC-F are not essentially tools for identifying patients with malnutrition and sarcopenia, but rather for identifying patients with deficient physical performance, i.e., a primary or secondary dysmobility phenotype, which may be due to sarcopenia, malnutrition or some other cause.

By now, screening for sarcopenia and malnutrition is not generally established in clinical routine care for osteoporosis patients, and accordingly, harmonized strategies and large-scale randomized trials of how to approach that additional risk are missing. However, there is growing evidence regarding beneficial effects of nutritional intervention and specifically high-intensity exercise programs on bone health [17,40,41,42].

While actual malnutrition and confirmed sarcopenia represent two tips of what can be considered an iceberg underneath the surface, MNA-SF and SARC-F are suitable tools to fathom its base, which is clinically best characterized by compromised physical performance long before the alarming loss of muscle mass and constitutional decline become obvious at the surface. Adhering to that picture, tracking this base of the iceberg and navigating fracture-preventive treatment accordingly appears advisable. In that regard, data and experience from this study confirm that both SARC-F and MNA-SF can easily be implemented in an osteoporosis routine care setting and provide additional and complementary information to identify patients at risk who putatively require additional treatment approaches beyond the prescription of bone-targeted drugs. Depending on individual risk profiles, this may include exercise interventions, nutritional counseling and supplementation.

## 5. Conclusions

SARC-F and MNA-SF do not specifically identify patients with sarcopenia and malnutrition, respectively, but including these questionnaires into the routine assessment of osteoporosis patients may be helpful to identify patients with impaired physical performance. Further studies should be done to evaluate screening tools for sarcopenia and malnutrition in osteoporosis patients.

## Figures and Tables

**Table 1:** Baseline characteristics of the study cohort with differences between patients with ≥4 SARC-F and ≤11 MNA-SF scores.

	All *n* = 107 (100%)	SARC-F ≥ 4 *n* = 33 (30.8%)	SARC-F < 4 *n* = 73 (68.2%)	*p*-Value °	MNA-SF ≤11 *n* = 38 (35.5%)	MNA-SF >11 *n* = 69 (64.5%)	*p*-Value *
Age (years)	75.0 (5.9)	77.1 (5.5)	74.1 (5.9)	0.014	76.6 (5.8)	74.1 (5.9)	0.035
Height (cm)	158.8 (6.0)	159.8 (5.0)	158.4 (6.4)	0.267	159.6 (6.2)	158.4 (5.9)	0.321
Weight (kg)	64.6 (11.9)	67.2 (12.6)	63.0 (10.8)	0.085	61.6 (14.5)	66.1 (9.9)	0.094
BMI (kg/m^2^)	25.6 (4.4)	26.4 (5.6)	25.1 (3.6)	0.215	24.1 (5.1)	26.4 (3.8)	0.010
SMI (kg/m^2^)	7.0 (1.2)	7.2 (1.3)	7.0 (1.2)	0.336	7.0 (1.2)	7.1 (1.3)	0.701
SPPB (points)	9.9 (2.6)	7.0 (3.1)	10.9 (1.4)	<0.001	8.7 (3.2)	10.5 (2.0)	0.005
CRT (s)	12.5 (5.2)	17.4 (6.9)	11.4 (4.0)	0.004	12.9 (5.7)	12.4 (4.9)	0.673
Usual Gait Speed (m/s)	1.2 (0.3)	0.9 (0.3)	1.4 (0.3)	<0.001	1.1 (0.3)	1.3 (0.3)	0.003
Handgrip Strength (kg)	20.2 (4.1)	17.4 (4.4)	21.2 (3.6)	<0.001	19.5 (4.4)	20.5 (4.0)	0.247
SARC-F (points)	2.7 (2.8)	6.3 (1.9)	1.0 (1.1)	<0.001	3.8 (3.3)	2.0 (2.3)	0.006
MNA-SF (points)	11.8 (2.3)	11.1 (2.5)	12.2 (2.1)	0.023	9.3 (1.7)	13.2 (0.9)	<0.001
Lumbar T-Score	−1.9 (1.5)	−1.5 (1.7)	−2.0 (1.3)	0.131	−1.6 (1.4)	−2.0 (1.5)	0.357
Femoral T-Score	−2.0 (0.9)	−1.8 (0.9)	−2.0 (0.9)	0.288	−2.2 (0.9)	−1.8 (0.9)	0.070
Femoral Neck T-Score	−2.4 (1.0)	−2.5 (1.2)	−2.3 (1.0)	0.607	−2.7 (1.2)	−2.2 (0.9)	0.108
Vitamin D (20–40 ng/mL)	38.7 (14.3)	36.3 (10.7)	40.2 (15.9)	0.225	37.4 (9.2)	39.5 (16.5)	0.444

° *p*-values for differences between patients with ≥4 SARC-F and <4 SARC-F scores. * *p*-values for differences between patients with ≤11 MNA-SF and >11 MNA-SF scores.

## Data Availability

All data and study material are stored at the Department of Orthopedics at the University of Würzburg for 10 years. The datasets used and analyzed during the current study are available from the corresponding author on reasonable request.
